# Metal Ion-Doped Hydroxyapatite-Based Materials for Bone Defect Restoration

**DOI:** 10.3390/bioengineering10121367

**Published:** 2023-11-28

**Authors:** Xuan Wang, Shan Huang, Qian Peng

**Affiliations:** 1 Xiangya Stomatological Hospital, Central South University, Changsha 410008, China; wyyanwx@126.com; 2 Xiangya School of Stomatology, Central South University, Changsha 410008, China; 3 Changsha Health Vocational College, Changsha 410100, China; huangshan2022208@163.com

**Keywords:** bone defect, restoration, hydroxyapatite, metal ion doping, bioactivity

## Abstract

Hydroxyapatite (HA)-based materials are widely used in the bone defect restoration field due to their stable physical properties, good biocompatibility, and bone induction potential. To further improve their performance with extra functions such as antibacterial activity, various kinds of metal ion-doped HA-based materials have been proposed and synthesized. This paper offered a comprehensive review of metal ion-doped HA-based materials for bone defect restoration based on the introduction of the physicochemical characteristics of HA followed by the synthesis methods, properties, and applications of different kinds of metal ion (Ag^+^, Zn^2+^, Mg^2+^, Sr^2+^, Sm^3+^, and Ce^3+^)-doped HA-based materials. In addition, the underlying challenges for bone defect restoration using these materials and potential solutions were discussed.

## 1. Introduction

Owing to injury, tumor, infection, functional atrophy, traffic accident, and other reasons, bone may have an interrupt of continuity and integrity, called bone defect. Such a defect often causes bone disjunction, delayed healing or non-healing, and local dysfunction. It requires restoration using a few specific artificial materials which should maximally imitate the structure and composition of natural bone tissue mainly consisting of organic compounds (mostly collagens) and inorganic nano-HA crystals [1]. To repair the defect, bone restoration materials are often used in clinical practice. The principle of bone defect restoration materials is to induce and promote the proliferation, differentiation, and deposition of the bone matrix of bone cells in the bone defect by providing bioactive substances and scaffold structure [2]. These materials should have comparable properties to regular bones, incorporating high porosity, magnificent cytocompatibility, great degradability, reasonable mechanical quality, and upgraded capacities for mineralization and bone cell proliferation [3,4].

Bone restoration materials are mainly divided into three categories: metal-based bone restoration materials, non-metallic bone restoration materials, and polymer materials. Among the non-metallic bone restoration materials, hydroxyapatite (HA) has been extensively used in tissue engineering. It has a high osteo-inductivity and is capable of bonding to bone tissue [5]. Meanwhile, HA has great biological properties like biological compatibility. There is a consensus that HA can promote the adhesion, growth, and differentiation of osteoblasts or osteoblast-like cells and boost the growth of cells supporting bone tissue like ligament cells or epithelium. Additionally, HA can serve as a stem cell carrier to speed up the restoration process [6]. However, it has a few disadvantages, like insufficient mechanical strength, degradation resistance, and antibacterial ability, which restrict its wide applications, especially those in complex in vivo conditions. The apparent disadvantages of HA, such as low mechanical strength, restrict its wide application. To improve its performance, many methods for modifying HA have been proposed. For example, the ions of some metals, including titanium, magnesium, and silver, show great biological potentials as they commonly exist in the human body and participate in most metabolic reactions [7]. Doping with these metal ions can also bring some extra benefits like the antibacterial ability to ensure overall performance of HA [8,9]. 

To date, a detailed review of multiple metal ion-doped HA-based materials for bone defect restoration to cure bone defect is still lacking. Most relevant reviews focused on the applications of pure HA to bone defect restoration [10,11], nano-HA and its composites with inorganic oxides or organic substances (e.g., chitosan) in tissue engineering and regenerative medicine [12], or biphasic calcium phosphates composed of HA and tricalcium phosphate for bone restoration [13]. A few of them reviewed the synthesis of specific metal ion-doped Ca_3_P_2_ materials for fixing normal bone defects or the applications of metal ion-doped HA as the coating of titanium dental implants [14,15]. Except for several common metal ions, such as silver and zinc ions, other metal ions for modifying HA-based materials including samarium ions are usually neglected.

The purpose of this study was to provide an in-depth review of metal ion (Ag^+^, Zn^2+^, Mg^2+^, Sr^2+^, Sm^3+^, and Ce^3+^)-doped HA-based materials for bone restoration to cure bone defect by tracking the latest research developments in their synthesis, physicochemical characteristics, properties, and applications, along with a discussion on the effects of a number of factors affecting bone defect restoration, such as the potential cytotoxicity and higher mechanical strength induced by doping with some metal ions, and the challenges and corresponding promising solutions for improving the utilization of metal ion-doped HA-based materials in the field of bone defect restoration.

## 2. Basic Characteristics and Functions of HA in Osteogenesis

Natural HA crystal plays an important role in the chemical storage of calcium (Ca) and phosphorus (P) in vivo and has a crucial effect on bone tissue healing [7]. Artificial HA crystal is a kind of bio-ceramic material regarded as the major inorganic substitute for the alveolar bone because of its similar composition to natural HA crystal. HA has stable physical characteristics, great biocompatibility, and bone-induction ability [16,17,18]. HA can also act as a stem cell carrier to speed up the restoration. As an implant material, its abilities of bone induction and bone conduction are indispensable in bone defect restoration. The former is a chemical process that induces surrounding cells to differentiate into osteoblasts and create new bones, while the latter is a physical process in the matrix of the material which can form a micro-scaffold for cells to enter and create new bones. Meanwhile, its ability to resist infection is worth consideration. These properties make it helpful in the growth and differentiation of stem cells derived from fat or bone marrow mesenchymal cells (BMSCs) [10,11,12,13,19]. It was proved that HA had huge potential and bright prospect of application in orthopedics. The current theories on how HA affects bone defect restoration can be concluded as follows. Firstly, there is a ratio between its surface area and volume, which is proportional to the adhesion of bio-protein and bacteria. The suitable surface roughness is also positively related to cell adhesion. These features not only guide and offer sufficient conditions for the creeping of matrix and bone formation but also adjust degradation speed in vivo to ensure the stability of the material. Materials with a Ca/P ratio closer to that of natural bone tissues are preferred because they often possess better biological properties [11,12,13,14,15,20,21,22,23]. On the other hand, HA has low mechanical strength [14]. Additionally, it was reported that nano-HA might inhibit cellular growth and cause cytotoxicity with excessive usage [24]. There is an infection after surgery as the body’s immune system cannot protect the implant material from bacterial colonizing. Hence, it is essential to improve the properties of HA.

## 3. Metal Ion-Doped HA-Based Materials

**Figure 1:**
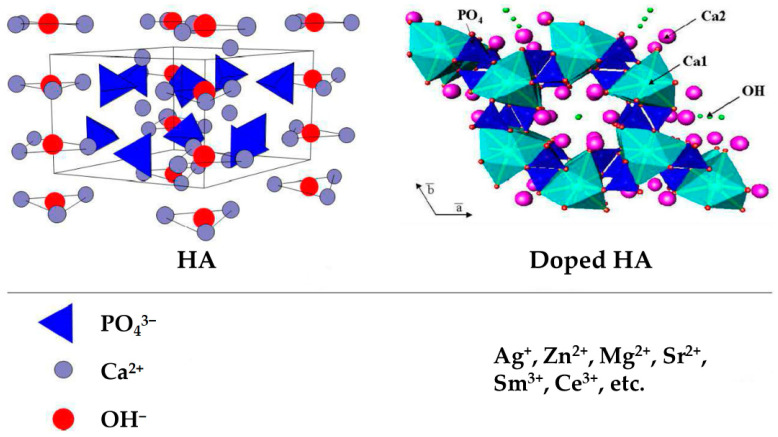
Structures of HA and doped HA. The unit cell of hydroxyapatite projected along a axis (**left**) and along c axis (**right**) showing tetrahedral phosphate, calcium and hydroxyl sites, with potential ionic substitutions (**bottom**). Adapted with permission from Ref. [28]. Copyright 2020, Daniel Arcos and María Vallet-Regí.

Combining with other materials via metal ion doping is an effective solution for HA to overcome its disadvantages. Metal ions, including those of silver (Ag^+^), zinc (Zn^2+^), magnesium (Mg^2+^), and strontium (Sr^2+^) can significantly affect the bone formation process, except anions, such as those of chlorine (Cl^−^), fluorine (F^−^), and carbonate (CO_3_^2−^) [25]. It is recognized that these metal ions play vital roles in a series of metabolism and physical functions in healing [26]. They alternate and occupy the vacant sites in HA [27]. Figure 1 shows the structures of HA and doped HA [28]. The structure of pure HA can be described as a hexagonal unit cell with space group P63/m and lattice parameters a = 9.432 Å and c = 6.881 Å, constituting Ca_10_(PO_4_)_6_(OH)_2_ per unit cell. The OH^−^ ions and four Ca^2+^ ions at Ca1 sites lie along columns parallel to the c axis. The OH^−^ is sited along the c axis, and the O–H bond direction is parallel to it, without straddling the mirror planes at z = 1/4 and 3/4. The remaining six Ca^2+^ ions, located at Ca_2_ sites, are associated with the two OH^−^ groups in the unit cell, where they form triangles perpendicular to the OH^−^ ions. The phosphate tetrahedrons form the remaining basic structural unit of HA. Some metal ions can replace Ca^2+^ with the valence of +2 inside HA crystal [29,30]. Because pure HA immersed in SBF has a negative charge due to the presence of hydroxyl (OH^−^) and phosphate (PO_4_^3−^) groups and metal ions show positive charge release from the surface, all charges can get neutralized and the HA material shows no electronegativity. When divalent Ca^2+^ ions are replaced by monovalent ions, fewer net positive charges exist in the “calcium-rich layer”, reducing the number of anions attracted to the electric double layer. At this moment, metal ion-doped HA has a positive charge [21]. The surface charge and specific surface area of metal ion-doped HA nanocrystals determine their differences in the capacity of an adsorbing protein, which also relies on the intrinsic properties of proteins in the medium [31]. Many biological proteins are negatively charged at pH 7.4. When the ions with a less positive charge, such as Ag^+^, are doped into HA crystals, a lower negative zeta potential weakens the electrostatic repulsion between Ag^+^-doped HA and negatively charged bio-protein, promoting the adsorption of proteins [21].

### 3.1. Synthesis Methods of Metal Ion-Doped HA-Based Materials

As shown in Table 1, the main synthesis methods of metal ion-doped HA-based materials are wet precipitation, sintering, and mechanochemical synthesis. 

Wet precipitation is a method of preparing materials via chemical reactions involving a liquid phase. One or several soluble metal salts or oxides (e.g., Ca(NO_3_)_2_·4H_2_O, (NH_4_)_2_HPO_4_, AgNO_3_, and Ca(OH)_2_) are used to prepare the solution so that each element is in an ionic or molecular state [21,23]. It is necessary to select the appropriate operations to enable precipitation or crystallization of the metal ions before the HA powder is obtained after treatment. This method is applicable because of its simplicity and low process cost. Low synthesis temperature (<100 °C) and harmless by-products (water) are some of other merits [41]. Another prominent characteristic of its products is low degradation, which increases the stability of doped materials [23,32,34]. However, its weakness is also obvious as no material produced by wet precipitation has shown improvement in mechanical performance. It indicates that wet precipitation offers little help in overcoming the inherent defect of HA. Regarding the heterogeneous reactions for synthesizing inorganic materials in aqueous media above ambient temperature and pressure, it is preferred to synthesize a broad range of nanometer materials. Homogeneous surface morphology could be observed [21]. However, it has the drawback of a relatively low yield of product [42].

Sintering is a method of producing bulky products by heating of solid powders of raw materials. To form ion-doped HA, chemical reagents, such as Ca(OH)_2_, H_3_PO4, CaHPO_4_, P_2_O_5_, Na_2_CO_3_ and CaF_2_, can be used to synthesize HA firstly. Some additives, e.g., PVP, TC, TEP and metal salts or oxides like Sr(NO_3_)_2_, Sm_2_O_3_ and Ce(NO_3_)_3_, are often involved [35,39]. The powders of solid materials will shrink and densify in the sintering process. There are different types of sintering. Among them, atmospheric sintering is the most common sintering method. It proceeds under the usual atmospheric pressure. The material sintered via this method can obtain high strength and good compactness for better applications in vivo with low cost [37,43]. Other sintering techniques include selective laser sintering (SLS), spark plasma sintering (SPS), pressure-less sintering (PS), and so on. All of them are affected by a few factors, such as sintering temperature, pressure, and atmosphere. Each method has its own advantages and disadvantages [44]. The specific sintering technique should be chosen based on the target applications.

Mechanochemical synthesis is a less commonly used method for preparing metal ion-doped HA-based materials. For example, Sr^2+^-doped HA has been prepared via milling of CaHPO_4_, Sr(OH)_2_, and CaO with a powder-to-ball weight ratio of 1/20 at a rotation speed of 1200 rpm for 30 min [38]. This method has the advantage of simple operation in spite of difficult impurity control. 

### 3.2. Properties and Applications of Metal Ion-Doped HA-Based Materials for Bone Defect Restoration

According to Table 1, adding metal ions may help to overcome the biggest weakness of HA, namely low mechanical strength, and reduce the postoperative infection risk. Through the specific synthetic method on raw materials, the acquired product with rough surface and good wettability allows cells or bioproteins to adhere more strongly. Lower degradation rate and percentage provide more time for the material to stay in place and work. It is known that the regeneration of bone tissue greatly relies on the biomolecules and bio-proteins. Some metal ions help attract more bioactive factors and motivate the proliferation, differentiation, and mineralization of stem cells. Plenty of experiments and reports have certified that metal ions can improve bioactivity or even bring some additional salutary impacts, such as antibacterial effect, drug-carrier, and so on.

#### 3.2.1. Ag^+^-Doped HA-Based Materials

Ag^+^-doped HA is often synthesized by wet precipitation [35]. By this method, silver ions replace calcium ions inside the HA crystal and facilitate the crystal growth. The material has good chemical properties and biological performance allowing it to adapt to the internal environment [37]. Its anti-bacterial ability plays well due to the characteristics of silver ions and proper ion release of the doped material. 

The degradation rate of Ag^+^-doped HA can greatly affect its service life in vivo. It relies on the environmental conditions like temperature and ambient pressure. Among the Ag^+^-doped HA samples produced through the precipitation method with different Ag^+^ additions at room temperature under atmospheric pressure, it could be considered as almost no degradation. The presence of Ag^+^ also contributed to lower corrosion rates of the samples compared to pure HA [45]. Additionally, the pH value, which varied between 6.55 and 7.40 in all measurements for the Ag^+^-doped HA groups, was suitable for the living system [46]. 

The cytotoxicity of Ag^+^-doped HA can be revealed by the release of Ag^+^ ions in the test area. It was reported that the concentration of Ag^+^ released from almost all Ag^+^-doped HA samples displayed no significant difference and it was lower than the set toxic value in human blood [21]. This observation indicated that Ag^+^-doped HA had little cytotoxicity on cells in a large range of concentration, and its potential toxicity could be avoided by controlling ion addition. Ag^+^-doped HA maintained good biocompatibility and worked well in vivo as well as pure HA [32]. Additionally, it could attract more proteins and act as a carrier to collect more biological factors (BFs) and stimulate the adhesion and release of BFs. By combining with silver ions, the doped material adsorbed a higher amount of bio-protein. After 25 d, the percentage of bone morphogenetic protein-2 (BMP-2) release was 86% [21,37]. The good bone induction ability is another reason why Ag^+^-doped HA popularizes in the applications. The alkaline phosphatase (ALP) quantification test verified that the silver ions obviously raised ALP activity and improved osteogenesis ability [23]. 

**Figure 2:**
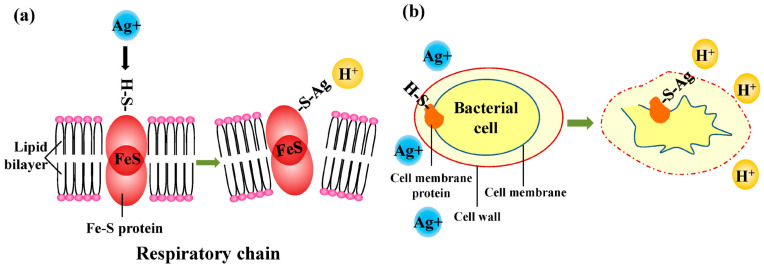
Antibacterial mechanism of Ag^+^ ions: (**a**) dysfunction of respiratory chain and membrane pumps and (**b**) shrinking and separation of cell membrane from cell wall, leaking cellular contents.

It was proved that combining with silver ions can bring an antibacterial activity which is beneficial for clinical application. The artificial Ag^+^-doped HA shows strong antibacterial activity, close to that of Ag^+^ ions. Its poisonous effects are mainly aimed at G^+^ bacteria. In a previous study, Ag^+^-doped HA was evaluated via bacteriological plate counting methods using G^−^ *E. coli* and G^+^ *S. aureus* [34]. Within an appropriate range, increasing the addition of Ag^+^ ions improved the bactericidal effect, which increased from 63% to 99% after incubation for 24 h and satisfied the National Standard of China GB/T 20944.3 protocol in which a percentage of bacteria reduction above 70% for *E. coli* and *S. aureus* is defined as the standard. The antimicrobial activity determined using a classical diffusion agar assay was also investigated [23]. The mixture of microorganism and material was cultured at 37 °C for 6 h. The viable bacteria count decreased to undetectable levels in 48 h for *E. coli* and in 96 h for *S. aureus*. The antimicrobial mechanism of Ag^+^ ions can be related to their interactions with thiolic groups (–SH) of proteins when the hydrogen atoms exchange with silver atoms, leading to the formation of S–Ag bonds (Figure 2). This reaction inactivates proteins in bacteria cells. Then, the cells denature and have changes such as dysfunction of the respiratory chain and membrane pumps. Additionally, the cell membrane shrinks and separates from the cell wall. As a result, the cell components leak out with the destruction of the cell wall. Another bactericidal mechanism is that the Ag^+^ ions may interact with DNA molecules, causing the molecules to condense to lose replication ability. The ions intensify the production of reactive oxygen species, enhancing the antibacterial properties. From the latter aspect, the Gram-negative bacteria are probably more susceptible to the action of silver ions due to a thin peptidoglycan wall, allowing increased accumulation of the ions in the periplasm [47]. The bacterium *Acinetobacter baumannii* is well known to everyone encountering hospital-acquired infections, including the ones of orthopedic implants such as prosthetic joints [48,49]. Silver ions have showed good resistance to *Acinetobacter baumannii* no matter whether working with other kinds of metal ions [50]. It is worth noting that a high concentration may bring stronger antibacterial activity but cause toxic effects in vivo at the same time. As a result, it is urgent to seek a proper concentration to maintain the balance between bactericidal activity and cytotoxicity.

#### 3.2.2. Zn^2+^-Doped HA-Based Materials

As a necessary trace element, zinc takes part in the metabolism of bone tissue as the active center in many types of enzymes and inhibits bone absorption. Zn^2+^-doped HA-based materials are mainly produced by wet precipitation, with the details shown in Table 1. They have good bioactivity and biocompatibility by showing a stimulative effect on the bio-protein expression and mineralization of osteoblasts. Zn^2+^-doped HA-based materials can also resist infection effectively with adjustable drug release [37]. 

The cell viability behavior and bio-protein expression of Zn^2+^-doped HA were proved to be significantly better than pure HA [29]. The mechanism of how zinc ions promote bioactivity can be concluded as follows. As an essential trace element for cell biosynthesis, zinc promoted protein synthesis as a protease catalyst, which was also the secondary messenger of a mitotic signaling pathway involved in regulating cell division [51]. Zn^2+^ ions released from the surface led to an increase in intracellular zinc concentration, which promoted fibroblast proliferation in oral system and protected those cells against oxidative stress and cell death [52]. 

According to a study, in the 6th month, many new bones formed in the central regions of a critical defect in the test groups, while in the pure HA group, there were few reborn connective tissues. In comparison with the pure HA group, Zn^2+^-doped HA had more connective tissues replaced by rebuilt bones, confirming that Zn^2+^-doped HA had a stronger osteo-induction potential [33]. For osteogenic differentiation and mineralization, increasing zinc concentration raised the expression of bone-specific transcription factor Runx2, a key inducer of osteogenic differentiation, osteocalcin, and ALP activity in the bone tissue micro-environment [53,54,55,56]. Moreover, Zn^2+^ ions hindered the transcription of gene NFATc1, inhibiting RAW264.7 osteoclastic differentiation in a dose-dependent manner [57,58]. The metal restrained osteoclasts by inhibiting the NF-κB pathway activation or by promoting osteoclast apoptosis [58]. In a word, Zn^2+^ ions act in promoting cell proliferation, increasing osteogenic activity, and inhibiting osteoclast bioactivity [59]. Zn^2+^-doped HA was considered as a suitable substitute for cancellous bone [60].

Like Ag^+^ ions, adding Zn^2+^ ions often endows the material with an antibacterial function. By choosing and incubating *S. aureus* at 37 °C for 1 d, 3 d, and 6 d to test the antimicrobial effect, it was demonstrated that *S. aureus* colonies were absent in the Zn^2+^-doped HA group on the 6th d. However, they still existed in the control group [29].

#### 3.2.3. Mg^2+^-Doped HA-Based Materials

As shown in Table 1, Mg^2+^-doped HA-based materials can be produced via sintering and precipitation. They are often featured by high density, porosity, and hardness. Their good wettability and low degradation rate make them suitable for application in vivo. As a bone implant material, it surely performs good biological properties.

Mg^2+^-doped HA has a similar structure to natural HA in bone through scanning electron microscopy (SEM) and transmission electron microscopy (TEM) [61]. Both processing technology and the content of metal ions can significantly affect the mechanical properties of the doped HA materials. According to the study of mechanical properties of Mg^2+^-doped HA scaffolds prepared via sintering [34], the mechanical strength relied on the process parameters such as sintering temperature and time. The maximum fracture toughness and hardness did not happen in the group with the highest dopant content but occurred in the groups with lower dopant contents. This phenomenon was probably because the changes in porosity and other mechanical characteristics did not follow the positive linearity. Instead, the higher addition may lead to a decrease in strength. After Mg^2+^ ions occupied part of the sites of Ca^2+^ ions, the changes in porosity and crystallinity inside the scaffolds contributed to bone tissue regeneration and obstruction of biodegradation in vivo. Moreover, the doping process could increase surface roughness, improving the bactericidal functions of the scaffolds. However, it does not mean that the higher porosity is better, especially when considering making the doped material able to load and release the drug. On the contrary, it may even lead to a reduction. This drawback could be partly mitigated by controlling the amount of Mg^2+^ ions properly.

The release of metal ions in Mg^2+^-doped HA scaffold was also explored by immersing all samples into phosphate-buffered solution (PBS) at 37 °C for 1, 4, 7, 10, and 14 d. It was confirmed that the release amount of Mg^2+^ ions was proportional to the amount of the doped Mg^2+^ ions. The release of ions can bring advantages like adjusting the degradation rate or adding some characteristics of Mg^2+^ itself. 

It was detected that by increasing doping concentration, cell growth and differentiation on the Mg^2+^-doped HA were accelerated. The great bioactivity of Mg^2+^-doped HA scaffolds can be proved as that the test groups had more cells adhering to their surface by spreading more filopodia and higher ALP activity, which embodies their great potential in inducing bone defect restoration. The release of Mg^2+^ ions efficiently built up an ionic microenvironment that significantly promoted early angiogenesis and osteogenesis within the bone defect area [62]. The ions could improve the infection resistance of HA, especially in its combined use with other metal ions [63,64].

#### 3.2.4. Sr^2+^-Doped HA-Based Materials

Sr is one of the most potent bioactive element candidates applied in biomedical engineering. Sr^2+^-doped HA can be produced via either wet precipitation or sintering, or even other methods. When applied in vivo, Sr^2+^-doped HA can reduce cytotoxicity [65]. This is because Sr^2+^ ions may restrain the dissolution of other metals by forming a competitive relationship of ion channels used to enter cells between Sr^2+^ and other metal ions [66]. In addition, Sr^2+^-doped HA has excellent biological properties by showing good bio-protein absorption or osteo-induction and acting as a drug loader [41]. 

The HA material with a high doping molar ratio of Sr^2+^ can significantly stimulate the growth of cells and promote their bioactivity. It was proved by a viable increment of viability from 25% up to 37% was observed after 7 d [36]. Apart from cell proliferation, the subsequent osteogenesis activity plays an important role in evaluating bioactivity for application. It is commonly agreed that the ALP activity, bone sialoprotein (BSP), and osteocalcin (OCN) can be used to determine the activity of osteogenesis and bone induction ability. The test indicated that cell differentiation might occur earlier in the material with high doping content of Sr^2+^. After the 21st d and 28^th^ d, the mineralization zone expanded in the test groups [30]. The data proved the improved bioactivity of the Sr^2+^-doped materials. Angiogenesis, well coupled with osteogenesis, plays an indispensable and vital role in modulating hard tissue regeneration. In fact, doping with Sr^2+^ showed a positive regulation effect towards osteogenesis [67,68,69,70,71,72].

Doping with Sr^2+^ ions allow HA to serve as a medicine carrier. Tetracycline (TC) is often selected as the model drug to measure drug loading efficiency and release profile. The antibiotic activity of TC in invitro study was assessed against Gram-positive bacteria *Staphylococcus aureus* and Gram-negative bacteria *Pseudomonas aeruginosa* [35]. The mSrHANFs material possessed excellent drug loading efficiency and released TC persistently to inhibit infection over three weeks. Additionally, either the polarization or the increased concentration of Sr^2+^ led to the antibacterial effect. With the extension of culture time, both Sr^2+^-doped HA and HA with higher Sr^2+^ doping concentration worked better in the late period, showing better antimicrobial performance [37]. 

#### 3.2.5. Other Metal Ion-Doped HA-Based Materials 

**Figure 3:**
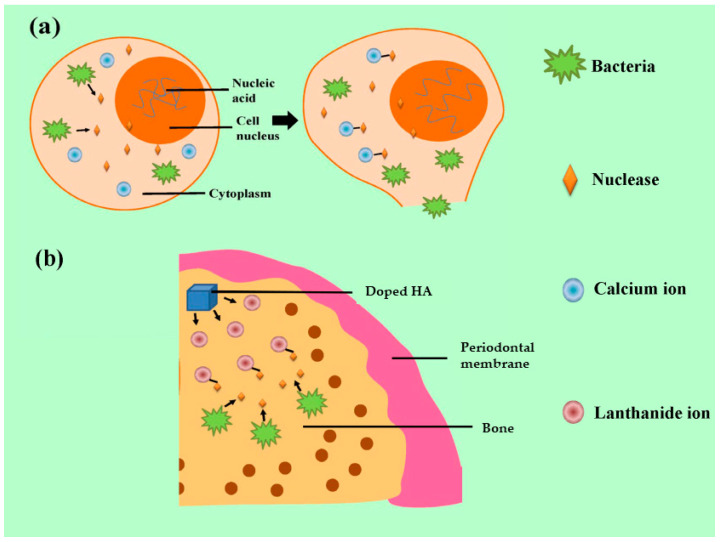
Antibacterial mechanism of lanthanide ions: (**a**) the pathogenic mechanism of nuclease released from *S. aureus* and (**b**) the antibacterial effect by combination of nuclease and lanthanide ions.

Lanthanide ions have similar ionic radii to Ca^2+^ ions and can replace Ca^2+^ ions in HA. The presence of these ions in the HA crystal structure appears to affect osteogenesis and osteoinduction [73,74,75,76,77,78,79]. Another important feature of lanthanides is their apparent antimicrobial properties which work against most bacteria [80,81]. For example, the antimicrobial mechanism of the ions is targeted at the nuclease of *Staphylococcus aureus*. The nuclease, regarded as one of the essential bacterial pathogenic factors, is dependent on the existence of Ca^2+^ ions and can degrade the nucleic acid to promote the spread of microorganisms [39,40]. A simple graphical representation is shown in Figure 3.

Adding Sm^3+^ could help overcoming the disadvantage of HA, low mechanical strength. The samarium ion (Sm^3+^)-doped P_2_O_5_ glass-reinforced HA materials (Sm^3+^-doped GR-HA) were successfully prepared [39]. The roughness of Sm^3+^-doped GR-HA decreased with increasing doping content. On the contrary, the density and bending strength increased in the test groups doped by Sm^3+^ ions compared to the group without Sm^3+^ ions (the control group). 

The biological properties, including cell adhesion, cell proliferation, and osteogenesis gene expression, were predicted by using MG63 cells to monitor osteogenesis based on the expressions of ALP, OCN, collagen-I (COL-I), BMP, and so on. The material surface was completely covered by a well-organized layer of elongated cells at the 7th d, presenting a perfect adaptation to the topography of the material. It was obvious that doping with Sm^3+^ could promote the expression of osteogenesis genes as all test groups had higher values of osteogenesis proteins than that of the control group. It was also reported that doping with Au^+^ exhibited a positive role in HA nucleation and growth [82]. It could promote cell formation [83]. Furthermore, doping with other ions, such as Ce^3+^, showed good osteogenesis activity with low cellular toxicity [84].

Gram-positive bacteria, like *Staphylococcus aureus*, *Staphylococcus epidermidis*, and *Pseudomonas aeruginosa* were used in the bacterial adhesion assay and showed less adhesion ability and lower activity. Compared to the control group, the adhesion of both *Staphylococcus* strains was greatly reduced in the Sm^3+^-doped GR-HA and positively depended on the Sm^3+^ concentration, which also appeared in the test of *Pseudomonas aeruginosa*. Similarly, the osteogenesis gene expression of Ce^3+^-doped GR-HA was higher than that of the GR-HA without the Ce^3+^ addition (the control group) [40]. Doping with Ce^3+^ ions also gave the material the antibacterial ability. The amount of G^+^ bacteria adhering to the surface of the material had a remarkable decrease compared to the control group. The bending strength, one of the main mechanical indicators, was higher in Ce^3+^-doped GR-HA than that in the control group. It proved that the doped material could perform good antibacterial effect. It was found that Ce^3+^ had the potential to work as a good carrier of ions and medicine [85].

Besides lanthanide ions, selenium, manganese, and gallium ions showed good potential for doping. It was proved that doping with them within suitable concentration ranges could provide good morphological and crystallographic features with tunable mechanical, antibacterial, and osteogenic properties [86,87].

## 4. Discussion

Owing to their great biocompatibility with many other beneficial effects, metal ions are prevalent in doping HA to produce metal ion-doped HA-based materials. These materials have a broad application prospect in restoring bone defect [88]. However, there are still some challenges, namely incomplete clarification of the mechanism of bone defect restoration, insufficient improvement of the properties in vitro, and rare in vivo tests, that need to be solved. 

Firstly, the mechanism of bone defect restoration using metal ion-doped HA-based materials has not been clarified completely because it depends on many factors, such as the type and content of dopant and the compositions and structures of the materials [21,23,35]. The roles of doped metal ions in improving bioactivity or osseointegration are associated with both the ions themselves and HA. It was reported that the layered structure of nano-HA deposited on the experimental material can provide many interval nano-cavities with a larger effective wetting area and higher hydrophilicity [89]. The improvement of hydrophilicity leads to easier dissolution of metal ions in the liquid internal environment to exert their impacts on cells [90], among which osteoblasts were the main targets. It indicates that other types of cells demand more attention. The existing studies demonstrated that most metal ions hardly contribute to solving the problem of the low mechanical strength of HA-based materials, which restricts the clinical application [91,92]. Until now, only a few reports have proved that specific ions, such as Sm^3+^, have good potential in improving the mechanical performance of HA with higher fracture strength [39]. It is possible that doping with metal ions can induce more grain boundaries which hinder plastic deformation of the material matrix, producing a strengthening effect. Moreover, it may cause twinning in the matrix, which introduces new twin boundaries that divide their host grains into smaller ones [93]. Consequently, there is an extra strengthening effect via grain refining. The other possible reasons for enhanced mechanical properties include the formation of a highly oriented crystalline structure due to the addition of metal ions [94]. It is essential to explore other kinds of metal ions. From this perspective, it is expected to be helpful to combine samarium ions and other metal ions together to improve mechanical properties of the materials with simultaneous retention of biological functions. 

Secondly, some verifications of improvement of the properties of metal ion-doped HA-based in vitro are still not sufficient and precise enough. For instance, it was found that the metal ion-doped HA coating produced via electric discharge sintering had an explosive release of Ag^+^ in the first 2 d, which can avoid the potential accumulation of toxicity. Moreover, the optimum concentration of each metal ion is essential in determining cytotoxicity, which has not been completely determined in the available reports. It is because many generic indexes for assessing poisonous effects are not standard and the related experiments are rare. The solution is to repeat the experiments with more specific values after determining a possible range. It has already been recognized that some kinds of metal ions can bring extra antibacterial effects to the HA-based material, and its mechanism and reaction duration had been reported [95]. However, most studies showed much better results on G^+^ bacteria. When acting on G^−^ bacteria, the bactericidal effects turned weak or even disappeared. It also indicated that some metal ions, such as Ce^3+^, may have potential benefits for G^−^ bacteria to grow [40]. It is probable that the adhesion ability of G^−^ bacteria is much stronger than G^+^ bacteria due to its outer membrane containing lipopolysaccharide and pilus, which enhance the adhesion ability. The two components also help them escape from exoteric interferences like metal ions or antimicrobial drugs [96]. The previous studies took only a few pathogens, such as *S. aureus* and *E. coli*, as experiment subjects. The long-term affections can be probed by testing more bacteria or prolonging the test duration. After the identification of the pathogenic mechanism, exploring new metal ion-doped HA-based materials loaded with targeted medicines should be put on the agenda. Besides mechanical properties, the cytotoxicity of metal ion-doped HA-based materials is another key point. Many related studies synthesized nanomaterials which can affect the differentiation process of reformed bone tissues [30]. However, the nanometer particles may enter cells and cause cytotoxicity. The cytotoxicity of metal ions is actually affected by many factors, such as metal ion size, dose, distribution in cells, and mode of entry. Among them, the ion size is deemed crucial to cytotoxicity. Like that of the ion dose [97], there is a negative correlation between ion size and toxicity in a certain range [98,99,100,101]. It may act via oxidative stress, leading to more intracellular reactive oxygen species (ROS) generation, cytokine production, and, ultimately, cell death. Additionally, the smaller ions will easily pass through the plasma membrane into the cell, which increases surface area and ion number for the same mass/volume dose, leading to amplified interactions with the cellular proteins and organelles [102]. In some cases, the ion size effect can be considered one of the main causes for determining dose-dependent properties [103]. Obviously, the effective measures for minimizing cytotoxicity include controlling the metal ion size, dose, and distribution. They are highly dependent on the material synthesis methods, which should be optimized.

Thirdly, there are rare in vivo tests certificating the suitability of metal ion-doped HA-based materials [104]. More efforts should be devoted to retention capacity in the in vivo application of implant materials. The abilities to control the degradation process and to resist corrosion are extremely important. Some metal ions, such as Ag^+^, may restrain the biological degradation process [32]. It is important to measure the working duration of the materials in animal tests and explore effects of different dose or concentration in vivo. Moreover, the cytotoxicity test should be boosted in animal tests. It is not viable to apply it clinically with just in vitro test results. Additionally, the practical bone restoration reaction should be further explored in real body to evaluate the comprehensive properties of the material.

Above all, it is essential to further explore the acting mechanism of the metal ions in HA and the combined role of different metal ions in controlling the performance of the doped products based on various examinations, especially the tests for functionalizing surfaces of metals to accelerate bone regeneration [105] and in vivo tests for specific clinic applications. 

## 5. Conclusions

This paper provides a systematic review of metal ion-doped HA-based materials for bone defect restoration covering the physicochemical characteristics of HA, synthesis methods, properties, and applications of different kinds of metal ion-doped HA-based materials, underlying challenges, and potential solutions. It was demonstrated that the materials could be used to solve problems in bone defect restoration. Especially, the metal ion-doped HA-based materials involving Ag^+^, Zn^2+^, Mg^2+^, or Sr^2+^ exhibited enhanced properties, including mechanical strength, corrosion resistance, and antibacterial activity, in comparison with pure HA. There are also some challenges restricting the development and use of metal ion-doped HA-based materials, including incomplete clarification of the mechanism of bone defect restoration, insufficient improvement of the properties in vitro, and rare in vivo tests. More efforts can be spent on enriching the mechanism of bone defect restoration, with further exploration of the properties in vitro and in vivo tests. Owing to their great potential for restoring bone defect, the materials will play a more active role in future clinical treatments by doping with multiple metal ions to make full use of their features. 

## Figures and Tables

**Table 1:** Synthesis and characteristics of metal ion-doped HA-based materials [21,23,29,30,32,33,34,35,36,37,38,39,40].

Metal Ion-Doped HA-Based Materials	Raw Materials	Synthesis Method	Experimental Conditions	Physical Features	Mechanical Properties	Chemical Properties	Biological Properties	Reference
Ultra-trace Ag^+^-doped HA	Ca(NO_3_)_2_·4H_2_O, (NH_4_)_2_HPO_4_, and AgNO_3_	Wet precipitation	Melting at pH 9.0 and room temperature, (Ag + Ca)/P molar ratio at 1.67, and Ag doping concentration between 0.27 ppm and 2.2 ppm	Clear and homogeneous surface morphology	/	Good Ag^+^ release ability: 46 ppb (Ag doping concentration of 2.2 ppm) and 11 ppb (Ag doping concentration of 0.27 ppm) after 48 h	Good protein absorption: 16.7 μg/mg (0.27 ppm) bovine serum albumin (BSA) at pH 7.4, over 15 μg/mg (2.2 ppm) BSA at pH 7.4; great antibacterial rate: 82% (0.27 ppm) and 90% (2.2 ppm) after culturing for 24 h; no cytotoxicity	[21]
Scaffold containing Ag^+^-doped HA	Ca(OH)_2_ and AgNO_3_	Wet precipitation	Stirring at 400 rpm for 1 h at 90 °C and Ag doping content at 5 mol.%	/	/	Low degradation rate; cumulative element release: near 1.0 mg/L at the 30th day	Great antibacterial effect: reducing to unviable count after 48 h for Escherichia coli (*E. coli*) and 96 h for *Staphylococcus aureus* (*S. aureus*); low cytotoxicity and good bioactivity; great bone restoration activity: alkaline phosphatase (ALP) content of over 0.10 per ng DNA	[23]
Ag^+^-doped HA	Ca(OH)_2_, H_3_PO_4_, and AgNO_3_	Wet precipitation	Stirring at pH above 10.5 at room temperature, sterilizing by autoclaving or heating at 1150 °C in air for 2 h, and Ag doping content of 0.5 wt.%	/	/	Low degradation rate and ion release: less than 0.5 ppm of Ag^+^ ions after immersion for 14 d and less than 1% of added Ag totally	Good antibacterial effect: almost no viable *S. aureus* colony survival from the starting population of 10^7^ CFU/mL; little cytotoxicity and great bioactivity	[32]
Zn^2+^-doped HA	Ca(OH)_2_, H_3_PO_4_, and Zn(NO_3_)_2_·6H_2_O	Wet precipitation	Stirring for 18 h with pH above 10.5, autoclaving at 124 °C for 2 h, and Zn doping content of 1.6 wt.%	/	/	/	Great bioactivity and bio-protein expression; effective antibacterial ability: almost no viable *S. aureus* colony survival from the starting population of 4 × 10^6^ CFU/mL	[29]
Zinc-containing calcium alginate-HA	(NH_4_)_2_HPO_4_, Ca(NO_3_)_2_·4H_2_O, and Zn(NO_3_)_2_ ·6H_2_O	Wet precipitation	Stirring for 3 h at pH 9 and 90 °C and Zn doping content of 0.5 wt.%	/	/	/	Good bio-compatibility: almost 100% cell viability after 24 h; great healing guidance: formation of almost 20% new bone after 6 months	[33]
HA-MgO scaffold	Natural HA, MgO nanoparticles, NaCl, and GN polymer	Sintering	Pressing at 120–160 MPa for 2 min and sintering at 1100–1150 °C with the addition of MgO of 10 wt.% or 15 wt.%	Uniform and dense architecture; rough surface; high density: near 2 g/cm^3^; high porosity: over 80%	Good elastic modulus: about 100 MPa; high hardness: about 60 N; good compressive strength: over 1.2 MPa	Excellent wettability; low degradation rate: less than 0.4%; using the same quantity of ions (1.5 mM) as that in human plasma	Good antibacterial behavior: nearly 60%; good biocompatibility	[34]
Polycaprolactone (PCL) membranes containing Sr^2+^-doped HA nanofibers (SrHANF)	TEP, pluronic P123, PVP, PCL, Ca(NO_3_)_2_·4H_2_O, Sr(NO_3_)_2_, and cetyltrimethylammonium bromide (CTAB)	Sintering	Operation with steady flowrate of 1.27 mL/h and electrical field of 1.3 kV/cm, calcining at 800 °C under a nitrogen atmosphere, Sr/(Sr + Ca) at 30 mol%, and (Sr + Ca)/P molar ratio at 1.67	/	/	/	Good osteogenic guidance and gene expression: MTT assay over 2.5 and ALP/MTT over 0.3 after incubating for 7 d	[30]
Sr^2+^-doped HA-CaO-CaCO_3_ nanofibers	Ca(NO_3_)_2_·4H_2_O, Sr(NO_3_)_2_, CTAB, pluronic P123, poly(vinyl pyrrolidone) (PVP) tetracycline hydrochloride (TC), and triethyl phosphite (TEP)	Sintering	Pressing under 120–160 MPa for 2 min, sintering at 1100–1150 °C, operation with steady flowrate (1.27 mL/h) and electrical field (1.3 kV/cm), calcining at 800 °C under a nitrogen atmosphere, Sr/(Sr + Ca) at 30 mol% and (Sr + Ca)/P molar ratio at 1.67	Big pore diameter: average pore diameter of 25.9 nm	/	Low degradation rate: 2.6% after immersion for 1d	High loading efficiency and slow release of drug: the amount of TC loaded at 97.21 ± 0.75% (*w*/*w*) and the release rate of TC at approximately 2.36% per day steadily	[35]
Sr^2+^-doped HA nanoparticles	Ca(NO_3_)_2_·4H_2_O, Sr(NO_3_)_2_, (NH_4_)_2_HPO_4_, and NH_4_OH	Wet precipitation	Stirring at 90 °C and pH 10.0 and Sr/(Sr + Ca) molar ratio at 1	Nanometer particles	/	/	Good biocompatibility and no apoptotic effect, great osteogenic bioactivity and guidance: an increment in MTT assay from 25% to 37% after 7 d	[36]
Polarized Sr^2+^-doped HA-barium strontium titanate (BST)	Sr^2+^-doped HA, BST, urea, and sodium polyacrylate	Sintering	Pressing under 100 MPa, sintering in air at 1250 °C for 2 h, polarizing at field intensity of 0.8 to 1.4 kV/mm for 30 min at 480 °C, and Sr doping content of 10 wt.%	/	Lower compressive strength (relative to pure HA): 23.4 MPa	/	Great osteogenic activity and gene expression: OD value between 2.5 and 3.0 after culturing for 14 d, relative expression of ALP at nearly 10 after culturing for 14 d	[37]
Sr^2+^-doped HA	CaHPO_4_, Sr(OH)_2_, and CaO	Mechanochemical synthesis	Milling of CaHPO_4_, Sr(OH)_2_, and CaO with the powder-to-ball weight ratio of 1/20 at a rotation speed of 1200 rpm for 30 min	/	/	/	Good cell adhesion and growth	[38]
Sm^3+^-doped glass-reinforced HA	CaHPO_4_, P_2_O_5_, Na_2_CO_3_, CaF_2_, and Sm_2_O_3_	Sintering	Sintering at 1300 °C under 80 MPa at a ramp rate of 4 °C/min for 1 h, Sm_2_O_3_/(Sm_2_O_3_ + P_2_O_5_) at 1 mol% and 2 mol%, CaO at 15 mol%, and CaF_2_ at 10 mol%	High density	High bending strength	/	Good bioactivity and osteogenic gene expression, and good antibacterial effect	[39]
Ce^3+^-doped glass-reinforced HA	Ca(OH)_2_, H_3_PO_4_, CaHPO_4_, P_2_O_5_, Na_2_CO_3_, CaF_2_, and Ce(NO_3_)_3_	Sintering	Sintering under 200 MPa at 1300 °C for 1 h (HA), sintering under 80 MPa at 1300 °C for 1 h using a heating rate of 2 °C/min (GR-HA), CaF_2_ at 10 mol%, Na_2_CO_3_ at 10 mol%, CaO at 15 mol%, P_2_O_5_ at 60 mol%, and CeO_2_ at 5 mol%	High surface roughness: Ra value of 6.37 ± 0.74 μm; low porosity (relative to pure HA): 10.96 ± 1.42% (Archimedes method) and 34.27 ± 0.34% (geometric method)	Low bending strength	Good hydrophilic ability: contact angle of 45.4 ± 1.6°	Great bioactivity and antibacterial effect (G^+^ bacteria)	[40]

## Data Availability

The data presented in this study are available on request from the corresponding author.
